# High Resolution Helium Ion Scanning Microscopy of the Rat Kidney

**DOI:** 10.1371/journal.pone.0057051

**Published:** 2013-03-07

**Authors:** William L. Rice, Alfred N. Van Hoek, Teodor G. Păunescu, Chuong Huynh, Bernhard Goetze, Bipin Singh, Larry Scipioni, Lewis A. Stern, Dennis Brown

**Affiliations:** 1 Center for Systems Biology, Program in Membrane Biology and Division of Nephrology, Department of Medicine, Massachusetts General Hospital and Harvard Medical School, Boston, Massachusetts, United States of America; 2 Carl Zeiss Microscopy, Peabody, Massachusetts, United States of America; Emory University, United States of America

## Abstract

Helium ion scanning microscopy is a novel imaging technology with the potential to provide sub-nanometer resolution images of uncoated biological tissues. So far, however, it has been used mainly in materials science applications. Here, we took advantage of helium ion microscopy to explore the epithelium of the rat kidney with unsurpassed image quality and detail. In addition, we evaluated different tissue preparation methods for their ability to preserve tissue architecture. We found that high contrast, high resolution imaging of the renal tubule surface is possible with a relatively simple processing procedure that consists of transcardial perfusion with aldehyde fixatives, vibratome tissue sectioning, tissue dehydration with graded methanol solutions and careful critical point drying. Coupled with the helium ion system, fine details such as membrane texture and membranous nanoprojections on the glomerular podocytes were visualized, and pores within the filtration slit diaphragm could be seen in much greater detail than in previous scanning EM studies. In the collecting duct, the extensive and striking apical microplicae of the intercalated cells were imaged without the shrunken or distorted appearance that is typical with conventional sample processing and scanning electron microscopy. Membrane depressions visible on principal cells suggest possible endo- or exocytotic events, and central cilia on these cells were imaged with remarkable preservation and clarity. We also demonstrate the use of colloidal gold probes for highlighting specific cell-surface proteins and find that 15 nm gold labels are practical and easily distinguishable, indicating that external labels of various sizes can be used to detect multiple targets in the same tissue. We conclude that this technology represents a technical breakthrough in imaging the topographical ultrastructure of animal tissues. Its use in future studies should allow the study of fine cellular details and provide significant advances in our understanding of cell surface structures and membrane organization.

## Introduction

Helium ion microscopy (HIM) is an imaging technology that uses a scanning beam of He^+^ ions to produce high quality images with the potential for sub-nanometer resolution. Such high resolution is made possible by the high brightness of the beam using a very small probe size, and the relatively short de Broglie wavelength of He^+^, enabling the beam to be focused to dimensions between 0.75 and ∼0.25 nm [1], [2]. As the He^+^ beam scans across the sample surface, liberated secondary electrons are collected, forming images of the sample surface topography. The classic choices for imaging biological samples have been: low voltage field emission scanning electron microscopy (LVFESEM), that can produce topographic images of samples with nanometer scale resolution, transmission electron microscopy (TEM) providing potential sub-nanometer resolution of thin tissue cross-sections, and atomic force microscopy (AFM) with sub-nanometer topographic resolution in all three dimensions, but with a limited depth of field. HIM offers a series of advantages compared to these imaging modalities: nanometer and sub-nanometer image resolutions, detailed surface topography and a high depth of field, all in uncoated samples so that surface details are not masked or obscured (for an in depth review of HIM image formation see [1]). In addition, while sample charging severely affects image quality in SEM imaging of uncoated biological samples, He^+^ ions are not deflected to the same degree as an electron beam, and the active charge neutralization of the Carl Zeiss Orion plus HIM mitigates these effects and preserves image quality.

Recently, studies have applied HIM to the evaluation of uncoated biological samples such as articular cartilage [3], colon cancer cells [4], platelet aggregation [5], and single cell surface topography [6], indicating that HIM has the potential to produce images of animal tissue that surpass what is currently achievable with electron microscopy. In this study we use HIM to explore the tubule epithelium of the rat kidney, with special attention to the impact of sample processing on sample integrity and image quality. In an attempt to minimize sample perturbation and maximize the preservation of the tissue architecture, we applied transcardial perfusion of aldehyde fixatives, and graded methanol dehydration prior to careful critical point drying (CPD), and we compare the preservation of overall and fine structural details of the tissues. We also explored the use of lectin gold and secondary antibody-gold conjugates as specific labels for highlighting specific components of the tubule surface membrane.

## Materials and Methods

### Tissue fixation

Animal experiments were approved by the Massachusetts General Hospital Subcommittee on Research Animal Care, in accordance with the National Institutes of Health, Department of Agriculture, and AAALAC requirements. 12 to 48 week old male Sprague Dawley rats were anesthetized with pentobarbital sodium (50 mg/kg body wt i.p, Nembutal, Abbott Laboratories, Abbott Park, IL) and perfused through the heart with phosphate-buffered saline (PBS, 0.9% NaCl in 10 mM phosphate buffer, pH 7.4) followed by paraformaldehyde (4%) lysine (75 mM) periodate (10 mM) fixative in 0.15 M sucrose, 37.5 mM sodium phosphate (modified PLP) as previously described [7], with a measured osmolarity of 935 mOsm/kg, or by 2 or 4% glutaraldehyde (GA) in 0.1 M sodium cacodylate (pH 7.4). The flow rate of perfusion was 20 ml/min and was performed for about 5–7 min with PBS and subsequently for about 5 min with the fixative. Tissues were post fixed overnight in modified PLP or GA at 4°C, washed in PBS, and stored at 4°C in PBS containing 0.02% NaN_3_.

### Vibratome sectioning

After fixation and removal from the animal, 500 µm thick tissue sections were cut under PBS using a TPI PELCO 101 series 1000 vibratome (Technical Products International, Inc., St. Louis, MO). The 500 µm thickness was chosen for ease of handling and to maintain the slice integrity during further processing steps. Slices were then returned to PBS containing 0.02% sodium azide at 4°C.

### External labeling

To label the surface glycocalyx of the kidney tissue, Triticum vulgare (WGA, EY Laboratories, San Mateo, CA) lectin (or agglutinin) conjugated to either 40 or 15 nm colloidal gold particles was diluted 1∶50 in PBS and incubated with the tissue overnight at 4°C, then washed three times in PBS. To label megalin on the PT cell membrane, the kidney tissue was incubated overnight at 4°C with a mouse monoclonal anti-megalin (1H2) primary antibody [8], [9] diluted 1∶100 in PBS, washed three times in PBS and then incubated with a 40 nm colloidal gold conjugated goat anti mouse IgG secondary antibody (EY Laboratories). After labeling, samples were washed thoroughly in PBS and post fixed for 2 h in 2% GA in 0.1 M sodium cacodylate (pH 7.4), and then washed again in PBS prior to methanol replacement and critical point drying.

### Methanol replacement

Kidney slices were placed into metal baskets and incubated with a mixture of 25% MeOH and 75% aqueous 0.1M (NH_4_)_2_CO_3_ for 2 hours at 2°C, while the temperature was lowered to 0°C using the Leica AFS freeze-substitution apparatus (Leica Microsystems Inc., Buffalo Grove, IL). The methanol-aqueous buffer was replaced twice with a fresh batch of 25/75 mixture, while the temperature was lowered to −10°C over a period of 4 h. A 40/60 methanol/aqueous buffer was employed two times over a period of 4 hrs, while the temperature was lowered to −20°C. It was followed by four 60/40 MeOH/aqueous mixture incubations over a period of 8 h while lowering the temperature to −40°C. We then used 80% MeOH in pure water for incubation (8 h) to drop the temperature to −60°C, followed by 100% MeOH (8 h, −80°C) and 100% MeOH (8 hrs, −90°C), replacing the MeOH at each step. Alternatively, a rapid series of graded methanol solutions in PBS was applied over a 4 h period at 4°C with the following schedule and MeOH dilutions: 25% for 60 min, 40% for 45 min, 60% for 45 min, 80% for 45 min, 100% for 45 min. For each gradation, the MeOH solution was refreshed halfway through the incubation.

### Critical point drying

Following the final MeOH replacement, the temperature of the samples in pure MeOH was raised to 0°C (at a rate of 3°C/min), the baskets were closed and placed into an Erlenmeyer flask containing ice-cold MeOH for transportation. Once MeOH and baskets were placed and secured in the critical point drying apparatus (Samdri-795, Tousimis Research Corp., Rockville, MD), the samples were purged with cold liquid CO_2_ (2°C) at elevated pressure, and then brought to supercritical pressure and temperature (1200 psi, 42°C) for incubation and equilibration (>4 min). The pressure was slowly reduced (<100 psi/min), while maintaining supercritical temperatures (>32°C), and after the bleeding process was completed, dried samples were mounted onto placeholders with sticky pads and stored under desiccant at room temperature. Storage under these conditions for more than one week did not result in obvious sample modifications.

### Helium ion microscopy

Helium ion microscopy (HIM) was carried out on an Orion helium ion microscope (Carl Zeiss Microscopy, Peabody, MA) at 35 keV beam energy, with a probe current ranging from 0.1 to 1.5 pA. No conductive coatings were applied to the samples prior to imaging, in order to preserve the sample surface information. Samples were transferred into the HIM via a load-lock system and were maintained at a vacuum of 2–3×10^−7^ torr during the imaging session. Charge control was maintained through the use of a low energy electron flood gun, which was applied in a temporally interlaced fashion with the imaging beam. Images were formed by collecting the secondary electrons elicited by the interaction between the helium ion beam and the sample with an Everhart-Thornley Microchannel plate [10]. This detector is also widely used in SEM, and consists of a scintillator placed inside a Faraday cage, that draws the elicited low voltage secondary electrons towards it. The resulting photons are collected, turned into electrons and amplified by a photomultiplier tube (PMT). The PMT signal is then digitized using an A/D converter and displayed as a grey value in a given pixel of the resulting image. The scanning of the helium ion beam and the formation of the image are synchronized so that for any given coordinate there is a corresponding signal or grey value in the resulting image. No post-processing procedures were applied to the digital images besides brightness and contrast adjustment [3]. The image signal was acquired in a line-averaging mode, with either 32 or 64 lines being integrated into each line in the final image. Charge neutralization was applied after each individual line pass of the beam.

### Scanning electron microscopy

Conventional scanning electron microscopy (SEM) was performed for comparison purposes. Wherever indicated, sputter coating was carried out at the Harvard University Center for Nanoscale Systems (Cambridge, MA) using a Cressington HR208 sputter coater (Cressington Scientific Instruments, Watford, England) and a Pt at 40 mA. The SEM was performed with a Merlin field emission scanning electron microscope (Carl Zeiss Microscopy) using either an SE2 or an in-lens detector.

### Measurement of cellular and cell membrane features

Images were imported into ImageJ software version 1.42q (NIH, Bethesda, MD), and the scale was set based on the scale bar in the annotated image file. To measure features in the image, the line bar was used, and measurements were expressed in micrometers. To measure pore size the line tool was used to determine the diameter of the pore at its largest axis.

The mean feature size and standard deviation from the sample mean were calculated in Excel version 12.3.4 (Microsoft Corp., Redmond, WA).

## Results and Discussion

### Tissue preparation

#### Fixation

For all high resolution imaging techniques, sample preparation is an important determinant of image quality [11]. Consequently, in the present study we compared the sample quality associated with various tissue fixation methods, previously reported to produce good results for electron microscopy studies, such as in situ fixation of kidney tissue by transcardial perfusion with either glutaraldehyde (GA) or modified paraformaldehyde lysine periodate (PLP). Formaldehyde-based PLP penetrates the tissue quickly, initiating the stabilization of tissue (defined as the initial protein-formaldehyde cross link), but cross-linking of proteins (formation of a methylene bridge between two proteins) is a slow process. In contrast, GA penetrates the tissue more slowly, but the chemical reaction to cross-link proteins is faster than for PLP [12]. Transcardial perfusion with either 4% GA or modified PLP, containing 4% paraformaldehyde, was used to preserve renal tubules such as proximal convoluted tubules (PT) and collecting ducts (CD) in a physiological, open conformation, which allows better visualization of the tubule lumen and apical surfaces of the epithelial cells.

#### Critical point drying (CPD)

CPD of tissues requires that tissue water is replaced by a suitable solvent that can be mixed with liquid carbon dioxide. However, little is known about the effects of methanol, acetone, or ethanol replacement on fixed tissue. Initially we systematically employed a lengthy series of graded methanol solutions while lowering the temperature to values above the freezing points of the methanol series to minimize sample–solvent interactions. Given the success of this procedure we found that a similar level of post CPD tissue quality was also achievable with a more rapid procedure using a graded methanol series at 4°C over approximately 4 h, indicating that a careful CPD protocol was important for tissue preservation. In this study, the kidney was sectioned into 500 µm thick sections to ensure ease of handling. The length of time needed for methanol to penetrate and replace the tissue water will vary with the tissue thickness, and methanol replacement is recommended for all samples. Indeed, even in thin samples consisting of only a few cells, a simple freeze drying procedure without methanol pre-treatment leads to poor tissue preservation [4]. The freeze-substitution/CPD technique was, therefore, used for the images shown here, unless otherwise stated.

### Glomerulus

**Figure 1:**
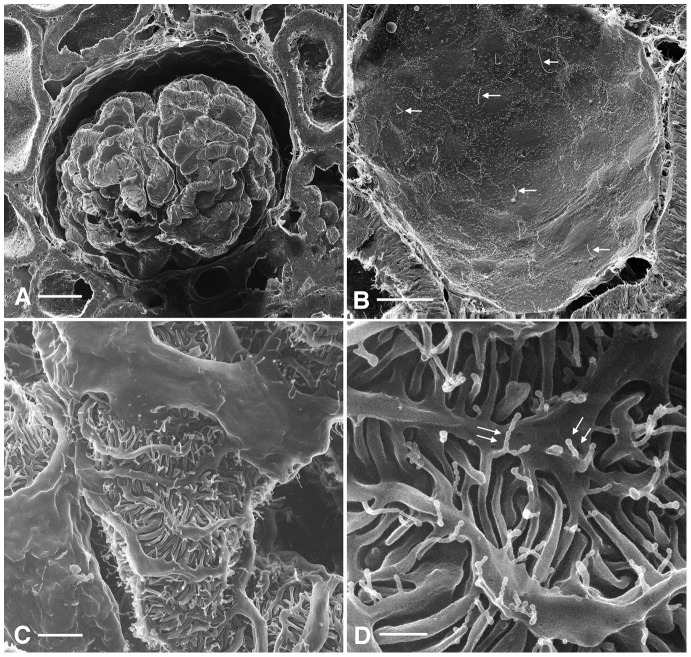
HIM imaging of kidney cortex.

Even at relatively low magnification, the high quality and depth of field of the HIM images is striking. Fig. 1A shows a whole glomerulus and neighboring tubules in the renal cortex. The branching processes of the podocytes surrounding the glomerular capillaries can be imaged at a resolution allowing the identification of fine features, such as the podocyte processes that envelop the capillaries. Fig. 1B shows the interior of a Bowman's capsule from which the glomerular capillaries (seen in Fig. 1A) were removed during the cutting process used to prepare the tissue. Long, single cilia project from each of the flat parietal epithelial cells that form Bowman's capsule.

**Figure 2:**
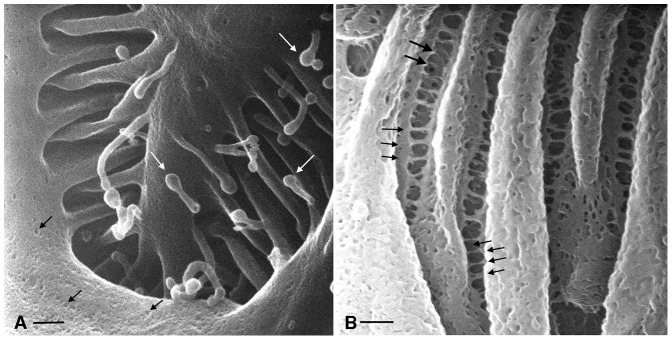
High magnification imaging of glomerular structures. (A) Detail of a glomerular podocyte showing a secondary projection and interdigitating foot processes (GA-fixation, extended methanol freeze-substitution dehydration protocol). Many tubular projections with more bulbous ends (white arrows) emerge from the podocyte membrane. Small (20–30 nm) irregularities of unknown nature can be seen on the external surface of the podocyte membrane (black arrows). Bar = 120 nm. (B) Detail of four “filtration” regions (slit diaphragms) between five adjacent podocyte foot processes. Numerous cross-bridging filaments extend at regular intervals across the space between adjacent foot processes (smaller arrows). In some regions, these delicate structures appear damaged, revealing another structure below, which may represent the glomerular basement membrane (larger arrows). Bar = 100 nm.

At a higher magnification (Fig. 1C) the complex interdigitations of the podocyte foot processes can be better appreciated. While these features have been described by conventional scanning EM in many studies [13], [14], the clarity of the HIM images and the specimen preparation method also allow clear visualization of numerous filamentous nano-protrusions originating from the major and minor processes and projecting into the urinary space (Fig. 1D). The width of these protrusions averaged 49.8±6.6 nm (mean ± SD, n = 26). At higher magnification, many of these protrusions had a bulbous end that was wider than the rest of the structure (Fig. 2A). However, in contrast to their paucity on podocytes from “normal” animals, longer filamentous projections have been described emerging from rodent podocytes that were subjected to injurious treatments such as puromycin [15] or cofilin depletion coupled with protamine sulfate exposure [16]. The role of these structures is unknown.

When imaged at an appropriate angle, membrane surface features in the form of 20–30 nm depressions were detectable on the podocyte plasma membrane. While these structures are reminiscent (in size and shape) of intramembrane particles, representing integral membrane proteins, that are visualized within the lipid bilayer by freeze-fracture electron microscopy [17], their nature is currently unknown. In well-oriented fields of view, a ladder-like structure was visible at the interface between adjacent foot processes, corresponding to the location of the podocyte filtration barrier (Fig. 2B). Throughout the glomerulus we measured pore widths with a mean of 22.0±8.0 nm (n = 12). A closer inspection of some filtration slits that possibly were damaged during tissue processing or perfusion provides a view of what may be the basal lamina below the podocytes (Fig. 2B). Recently Gagliardini and coworkers [18] described similar “slit diaphragm” structures by scanning EM of metal-coated samples using an “in-lens” detector to increase the sensitivity of the procedure. However, the HIM images offer the possibility of visualizing these structures at higher magnification than was previously achievable. Indeed the podocyte filtration slit is visible with remarkable clarity compared to that seen by Gagliardini et al. in their studies performed using LVFESEM [18]. The range in pore size dimensions measured in the current study are in good agreement with those described by Gagliardini et al., but the mean pore size we find in this study is 22 nm whereas they reported an average of 12 nm. This discrepancy may be due to the supraphysiologic perfusion fixation flow rate of 20 ml/min used in our study, leading to high arterial pressures compared to the flow rate used by Gagliardini et al., which was matched to the measured arterial pressure prior to fixation. However, we repeated the HIM study using kidneys that were fixed by immersion only and the results were essentially similar to our perfusion fixed tissues (data not shown). Another possible source for this discrepancy could be the difference in the dehydration method used in the two studies, given that Gagliardini et al. employs either dehydration in alcohols followed by CPD or dehydration in hexamethyldisilazane (HDMS) for their samples [18]. The different pore diameters measured in these studies could also possibly indicate that the filtration slit pores are dynamic, but more studies will be needed to examine this possibility.

**Figure 3:**
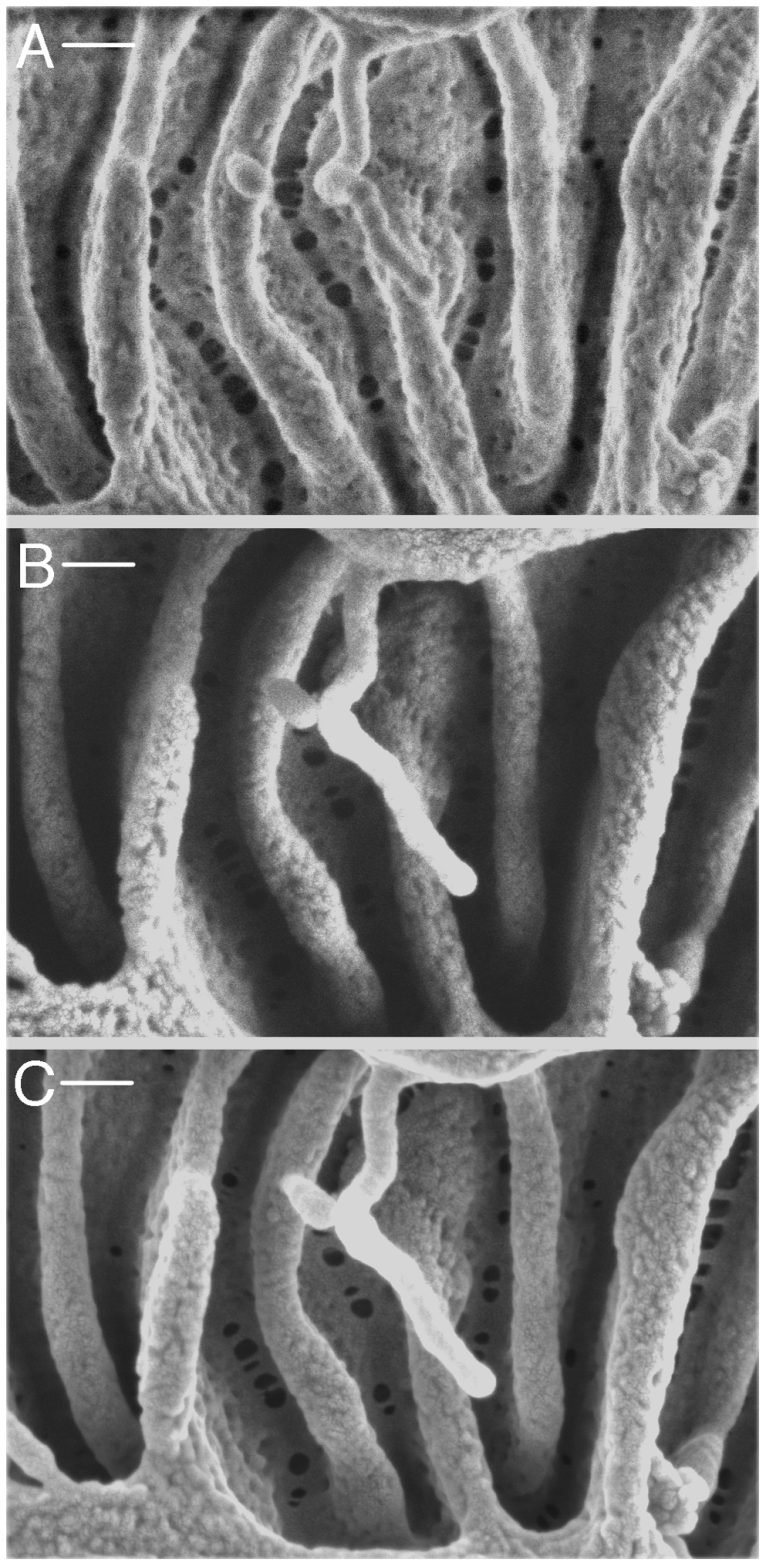
Glomerular podocyte slit diaphragms from the same kidney as shown by HIM in Fig. 2, imaged by conventional scanning electron microscopy (SEM). (A) Sample imaged without sputter coating, using an in-lens detector. (B, C) Coated samples imaged using either the standard SE2 detector (B) or an in-lens detector (C). Structural details of the slit diaphragm are less well defined than in the HIM image shown in Fig. 2B. Bar = 100 nm.

In order to establish that the quality of these high magnification images is due to the HIM technology and not to the tissue processing method we employed, we also imaged these tissues by conventional scanning electron microscopy (SEM) (Fig. 3). Fine structural details of the slit diaphragm are less well defined when imaged by SEM, both without (Fig. 3A) and with sputter coating, whether using the standard SE2 detector (Fig. 3B) or an in-lens detector (Fig. 3C).

**Figure 4:**
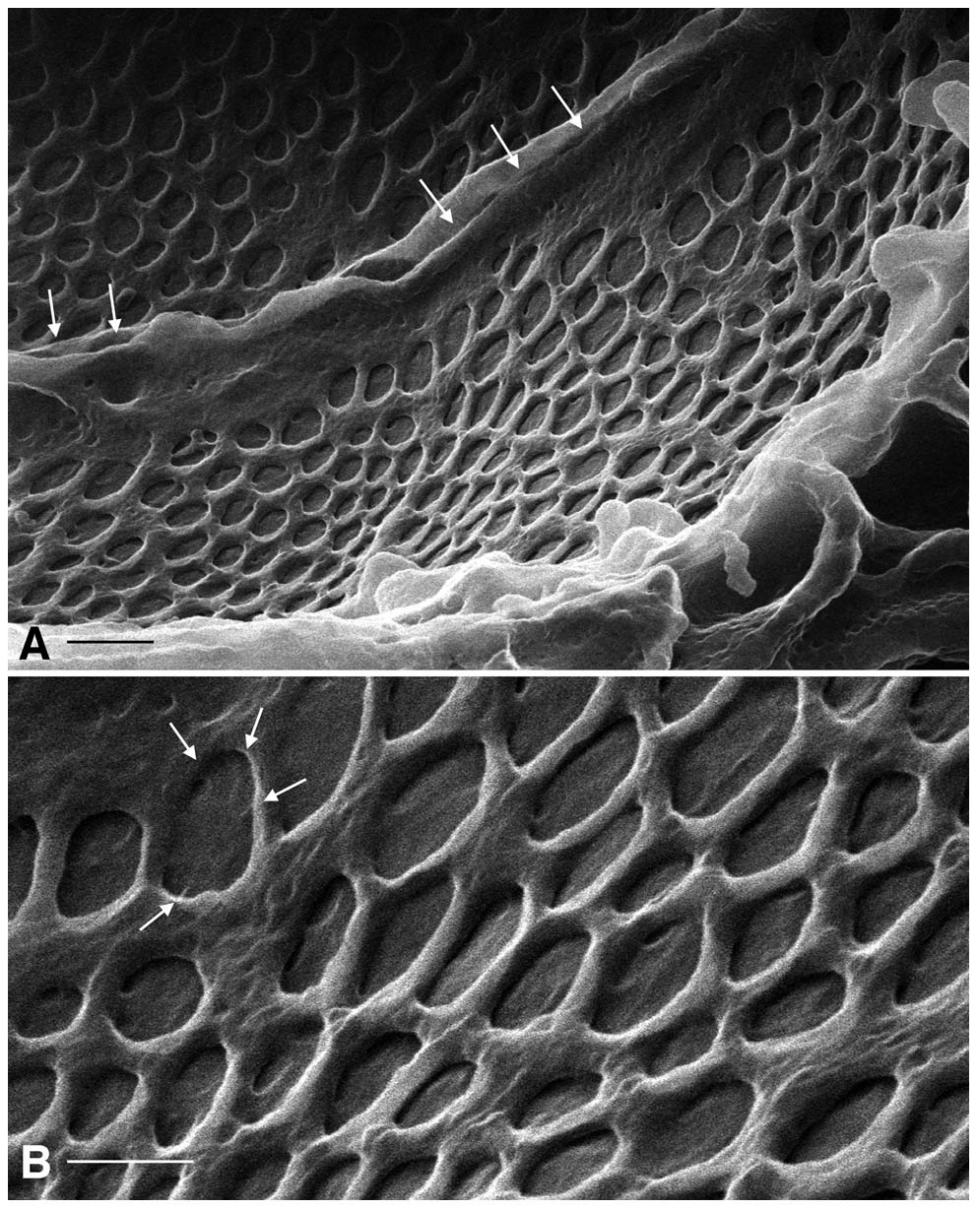
HIM imaging of glomerular endothelial cells. (A) Two adjacent endothelial cells from a glomerular capillary (GA-fixed, dehydrated using the extended methanol freeze-substitution protocol), imaged from the luminal side. The most striking features of these cells are the numerous, round fenestrations that are present over the entire cell surface. The raised ridges (arrows) represent the location of the tight junction between the two cells. Bar = 175 nm. (B) Higher magnification showing details of the fenestrations. In some of them, a substructure consisting of faint spokes like a bicycle wheel can be seen (arrows). Bar = 80 nm.

Beneath the podocytes and the basal lamina lie the endothelial cells of the glomerular capillary. Random cuts frequently expose the glomerular endothelium and this allows visualization of the cell surface (Fig. 4). The dominant feature of these endothelial cells are the numerous fenestrae with a diameter of 74.0±14.8 nm (mean ± SD, n = 35). Fig. 4B shows a radial patterning visible in the center of some fenestrae, which is similar to what has been described for the endothelial diaphragm (arrows) using a rapid-freeze, deep etching procedure for other fenestrated endothelial cells [19], although such structures were reported to be absent from the glomerular endothelium. Raised ridges corresponding to the location of the junction between adjacent endothelial cells can also be seen (Fig. 4A).

### Proximal tubule

**Figure 5:**
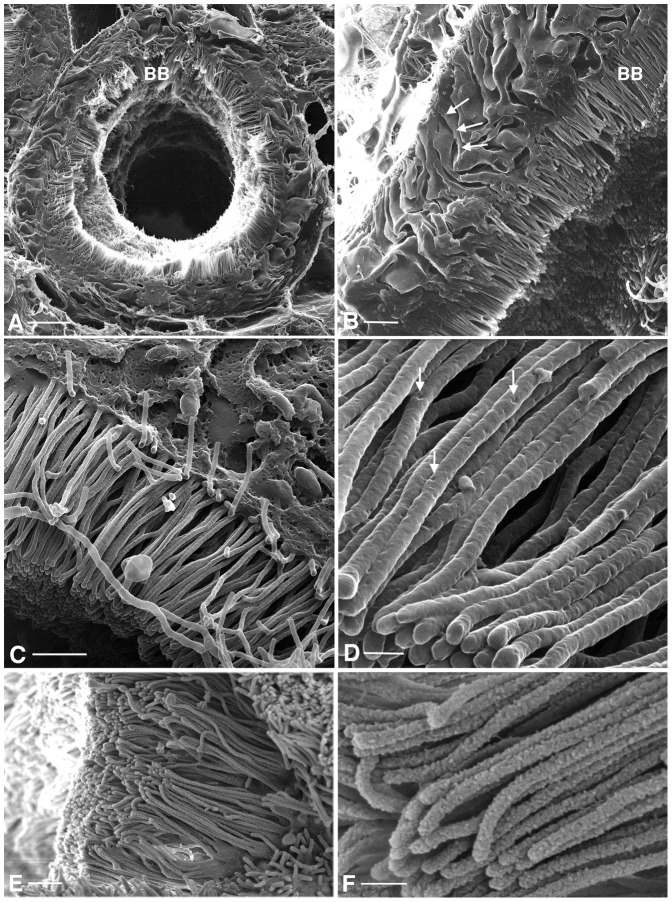
Imaging of renal proximal convoluted tubule. (A) Lower magnification showing GA-fixed proximal tubule (dehydrated using the extended methanol freeze-substitution protocol) and its extensive brush border (BB). Bar = 5 µm. (B) shows a lateral section of modified PLP-fixed proximal tubule dehydrated as in (A), demonstrating the apical brush border (BB) and the extensive basolateral plasma membrane infoldings and invaginations (arrows) that are characteristic of the S1 segment of the proximal tubule. Bar = 1 µm. (C) shows the tightly packed, slender brush border microvilli in greater detail (GA fixation, extended methanol freeze-substitution dehydration protocol). Bar = 0.5 µm. (D) Brush border microvilli at high magnification showing that their surface membrane has numerous micropits of unknown significance (arrows). Bar = 100 nm. Similar regions from the same kidney were also imaged by conventional SEM after coating using the in-lens detector and are shown at lower (E, bar = 0.5 µm) and higher magnification (F, bar = 100 nm). The conventional images have considerable less clarity and surface detail than the HIM-imaged brush border region.

Reabsorption of the ultrafiltrate begins with the proximal tubule (PT), which is characterized by a well-developed brush border that increases considerably the apical surface area of the tubule. This brush border often appears bright at low magnification in HIM (Fig. 5A), making the PT easily identifiable. The prominent brush border of the PT was evident with all fixation methods. Complex interdigitations of the lateral cellular membranes of proximal tubule cells were readily visualized (Fig. 5B). Figs. 5C, D show the long, slender structure of the brush border microvilli. These microvilli appear to be quite uniform in size, with a length of 2.73±0.13 µm (n = 10) and a diameter of 48.5±6.4 nm (n = 28). The microvilli can be imaged at very high magnification, where their surface appears pitted rather than uniformly smooth (Fig. 5D). As determined for the slit diaphragm (Fig. 3), the clarity and detail of the brush border images is superior by HIM imaging than by conventional SEM (Fig. 5E, F).

### Collecting duct

**Figure 6:**
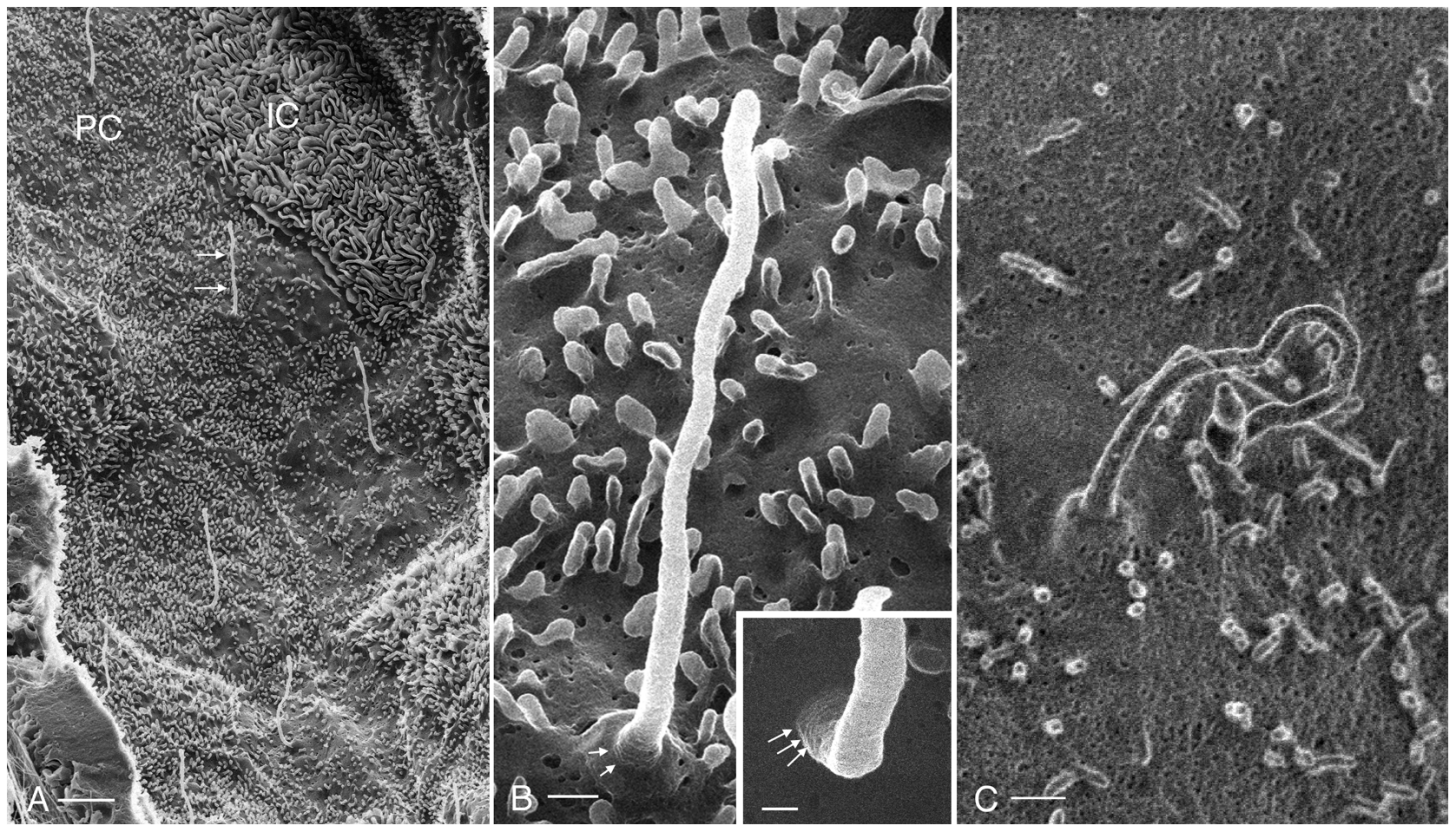
Imaging of renal collecting duct. (A) Luminal surface of an outer medullary collecting duct (GA-fixation, dehydration using the rapid graded methanol procedure) showing principal and intercalated cells. Each principal cell (PC) has one long, solitary cilium (arrows) and numerous short, stubby microvilli. The intercalated cell (IC) has numerous elaborate apical microplicae and no cilium. Bar = 2 µm. (B) High magnification view of a principal cell cilium (Bar = 200 nm). At its base, a concentric pattern of surface protrusions (arrows) can be seen in the position of the ciliary necklace. A similar structure is shown on another principal cell cilium in the inset (arrows, Bar = 100 nm). A principal cell cilium from the same kidney was also imaged by conventional SEM without sputter coating, using an in-lens detector (C). Structural details of the cilium, ciliary necklace, microvilli, and membrane indentations are more clearly distinguishable in the HIM than in the SEM images. Bar = 300 nm.

**Figure 7:**
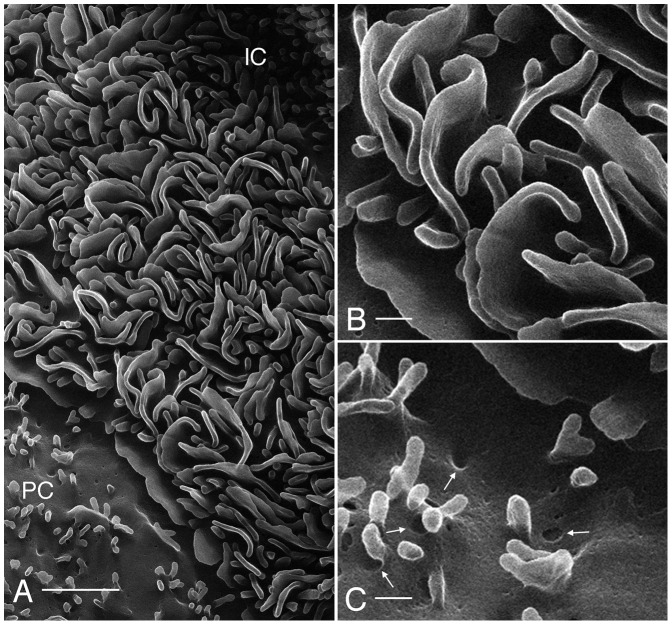
Detail from Fig. 6A showing the principal cell (PC) and an intercalated cell (IC) at higher magnification. The apical membrane of the intercalated cell has a highly complex organization that is formed of many microplicae and membrane furrows between these structures. Bar = 1 µm. (B) Higher magnification image of the elaborate intercalated cell apical membrane microplicae showing the deep infoldings of this membrane domain. Bar = 200 nm. (C) Apical membrane of a principal cell showing surface features that may represent exocytotic or endocytotic events. These depressions were frequently seen at the base of the short microvilli - a location in which clathrin mediated endocytosis often occurs. Bar = 200 nm.

The collecting duct (CD) is the main site for vasopressin-regulated water reabsorption in the kidney, and distal acid/base regulation, processes mediated by the principal and intercalated cells respectively [20]. HIM imaging reveals the surface architecture of the CD cells in great detail (Fig. 6A). Previous scanning EM studies have shown that the principal cells (PC) are clearly distinguishable from the intercalated cells (IC) based on structural features [13], [21]. The surface area of the PC is smoother than that of the IC and is populated by short, stubby microvilli (Figs. 6A, B), and all PC have a prominent solitary cilium measuring 2.90±0.32 µm (n = 13) in length, with a diameter of approximately 100 nm. In Fig. 6B and the inset, a closer inspection of PC cilia reveals ring-like structures at their base, possibly representing the ciliary necklace that has been described by freeze-fracture electron microscopy [22]. Details of the CD cell membrane structural features are more clearly defined by HIM than when SEM is used (Fig. 6C). Indentations in the apical plasma membrane of the PC may represent various configurations of endo- or exocytotic events (see also arrows in Fig. 7C) that are common in this membrane domain. Clathrin mediated endocytotic events frequently occur at the base of the microvilli [23], [24], [25], consistent with the membrane depressions highlighted in Fig. 7C.

In contrast to PC, the collecting duct IC can be identified based on their extensive apical microplicae and by the absence of cilia. IC can be activated by various agents via metabolic pathways, involving enzymes such as protein kinases A and C [7], [26]. When activated, IC not only significantly increase their rates of proton secretion into the CD lumen, but also undergo morphological modifications, corresponding to a noticeable elongation and increase in number of their apical microvilli or microplicae [7], [26], [27], [28], [29]. In Fig. 6A, an IC appears activated based on the number and length of its apical microplicae, which are shown in progressively greater detail in Figs. 7A, B, and in comparison with some other IC (data not shown) that typically exhibit fewer and shorter microplicae. The extensive infoldings and microplicae of the IC apical surface form very characteristic, channel-like passages adjacent to the membrane folds that could represent a microdomain of specific ionic composition and pH, and which plays a role in the specialized proton-secreting function of these cells. This unusual membrane configuration is quite different from the usual type of membrane amplification seen in other cells, including proximal tubule brush borders and principal cell microvilli.

### External gold labeling

**Figure 8:**
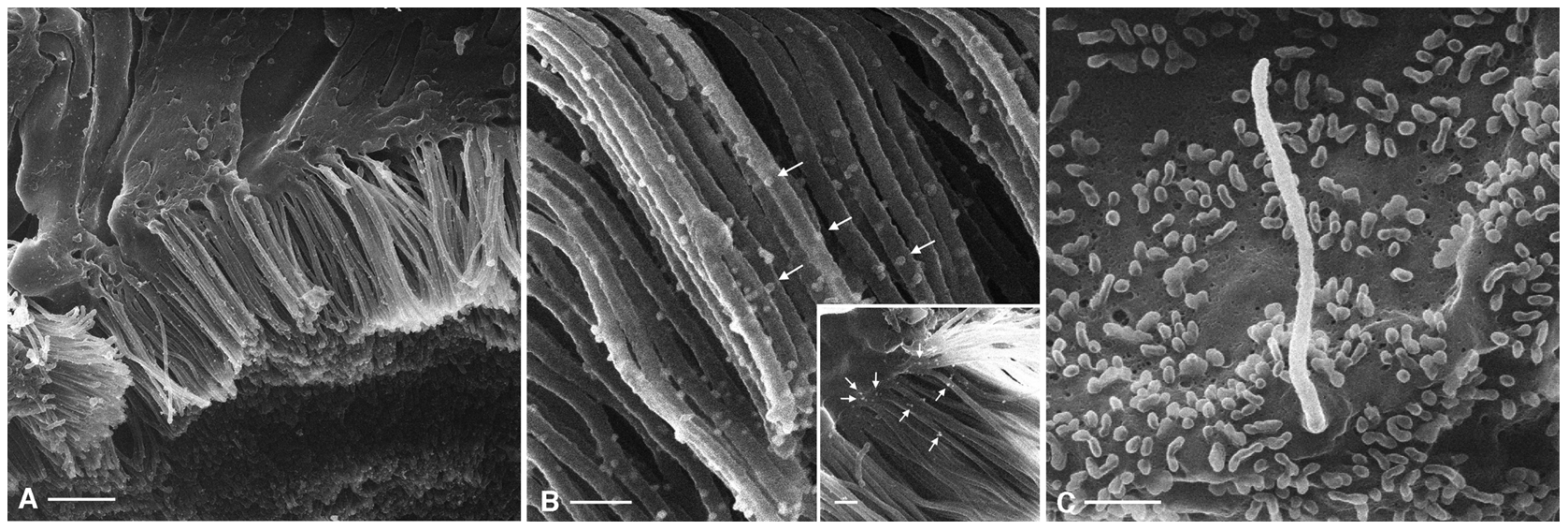
HIM imaging of external gold labeling in the kidney. (A) Lower magnification of a modified PLP-fixed proximal tubule with its brush border after labeling of surface glycoproteins (and/or glycolipids) with gold-conjugated WGA. The tissue was dehydrated using the rapid graded methanol procedure. The gold particles appear as discrete, white globular entities associated with the external surface of brush border microvilli and other parts of the cell surface adjacent to the microvilli. Bar = 1 µm. The gold label can be seen more easily at higher magnification (B - arrows), where it extends along the entire length of the microvilli. Bar = 200 nm. The inset in panel B shows a modified PLP-fixed proximal tubule brush border that has been immunolabeled with a monoclonal anti-megalin antibody followed by a secondary, gold-conjugated anti-mouse antibody. In this case, the pale gold particles (arrows) are concentrated towards the base of the microvilli and do not extend along their entire length (inset; Bar = 200 nm). (C) The apical surface of a collecting duct principal cell from the same kidney immunolabeled with the anti-megalin antibody and the respective gold-conjugated secondary antibody. The image shows no gold particles, attesting to the specificity of the proximal tubule megalin binding. Bar = 500 nm.

Besides imaging uncoated samples at high resolution, we were able to use colloidal gold probes to label tissue samples without a separate detector and with no additional enhancement. Immunogold labeling has previously been applied using conventional scanning microscopy but optimal visualization requires the use of secondary electron imaging (SEI) and EDS (energy dispersive X-ray microanalyzer) [30]. Combining the SEI mode with backscattered electron imaging (BEI) can also provide a correlation between gold labeling and surface topography [31], [32], but with considerably less surface detail that is possible with HIM. The SEI mode alone can also be used if gold particles are revealed by silver enhancement, but this requires subsequent post-acquisition image analysis to provide images of acceptable quality [33]. Because we can easily visualize and determine the size of the gold labels morphologically using HIM, it will now be possible to detect the association of multiple antigens with cellular structures in the same sample. As an example of gold labeling, we chose the previously-described proximal tubule marker gp330/megalin [34] and Triticum vulgare (wheat germ) agglutinin (WGA), which labels PT cell membranes in addition to other cell types in the kidney [35], [36]. The membrane of PT cells, including the apical microvilli, can be labeled with WGA conjugated to 26 nm colloidal gold (commercially available as 40 nm gold-conjugated lectin, but having an actual measured size of 26 nm, as determined through HIM) (Figs. 8A, B) or with a megalin primary antibody and a 15 nm gold conjugated secondary antibody (Fig. 8B, inset). The gold particles are easily distinguishable as bright spheres at higher magnification, indicating the suitability of HIM for uncovering the spatial distribution of antigens on the membrane surface. Indeed, while the WGA-gold probe labels the entire length of the microvillar surface, the megalin-gold label is more concentrated towards the base of the microvilli, as previously described using conventional, thin section immunostaining [9], [34], [37]. The lack of megalin-associated gold labeling in the collecting duct cells further attests to the specificity of the immunostaining (Fig. 8C).

The relatively pale appearance of the gold particles (distinct from their dark, electron dense appearance in transmission EM) does make it somewhat difficult to examine tissues rapidly at low magnification. A probe yielding a greater degree of contrast with respect to the surrounding tissue would, therefore, be preferable. He ion–sample interactions can produce sample fluorescence through the cathodoluminescence effect [2], [6]. Since quantum dots are approximately 11 nm in size, this would make them an interesting possibility for dual fluorescence/size HIM contrast labels. These and other alternative probes for immunolabeling specimens will be tested in future studies.

Typically, high resolution, topographical imaging of biological specimens with SEM often requires low voltage high-resolution field-emission SEM [11] to achieve sub 5 nm resolution in coated samples. Imaging of uncoated samples, while ideal for preserving the fidelity of the tissue architecture, presents challenges for SEM due to sample charging artifacts that are easily mitigated in HIM. Here, we have presented images from uncoated biological specimens with resolutions in the 2–5 nm range without complicated tissue preparation. The highest resolution images were taken at a digital resolution of 0.48 nm per pixel, resulting in a theoretical resolution of 1.4 nm.
